# Precursor-Led Grain Boundary Engineering for Superior Thermoelectric Performance in Niobium Strontium Titanate

**DOI:** 10.1021/acsami.2c22712

**Published:** 2023-02-28

**Authors:** Yibing Zhu, Feridoon Azough, Xiaodong Liu, Xiangli Zhong, Minghao Zhao, Kalliope Margaronis, Sohini Kar-Narayan, Ian Kinloch, David J. Lewis, Robert Freer

**Affiliations:** † Department of Materials, School of Natural Sciences, University of Manchester, Manchester M13 9PL, U.K.; ‡ Department of Chemistry, School of Natural Sciences, University of Manchester, Manchester M13 9PL, U.K.; § Department of Materials Science & Metallurgy, University of Cambridge, 27 Charles Babbage Road, Cambridge CB3 0FS, U.K.; ∥ Henry Royce Institute and National Graphene Institute, University of Manchester, Oxford Road, Manchester M13 9PL, U.K.

**Keywords:** thermoelectric, strontium titanate, high charge mobility, MoS_2_, grain boundary resistance, ammonium tetrathiomolybdate

## Abstract

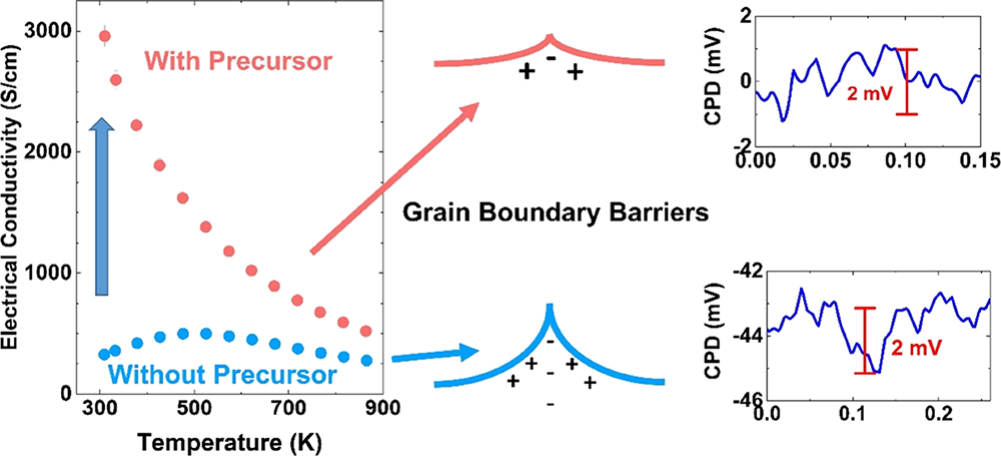

We present a novel method to significantly enhance the thermoelectric performance of ceramics in the model system SrTi_0.85_Nb_0.15_O_3_ through the use of the precursor ammonium tetrathiomolybdate (0.5–2% w/w additions). After sintering the precursor-infused green body at 1700 K for 24 h in 5% H_2_/Ar, single-crystal-like electron transport behavior developed with electrical conductivity reaching ∼3000 S/cm at ∼300 K, almost a magnitude higher than that in the control sample. During processing, the precursor transformed into MoS_2_, then into MoO_*x*_, and finally into Mo particles. This limited grain growth promoted secondary phase generation but importantly helped to reduce the grain boundary barriers. Samples prepared with additions of the precursor exhibited vastly increased electrical conductivity, without significant impact on Seebeck coefficients giving rise to high power factor values of 1760 μW/mK^2^ at ∼300 K and a maximum thermoelectric figure-of-merit *zT* of 0.24 at 823 K. This processing strategy provides a simple method to achieve high charge mobility in polycrystalline titanate and related materials and with the potential to create “phonon-glass-electron-crystal” oxide thermoelectric materials.

## 1 Introduction

Thermoelectric (TE) materials can convert waste heat into electrical power, providing an important energy-harvesting approach for sustainable development.^1^ The dimensionless, TE figure-of-merit *zT* can be calculated via eq 1 and is used to describe TE performance and assess candidate materials,
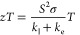
1where *S*, σ, *T*, *k*_l_, and *k*_e_ refer to the Seebeck coefficient, electrical conductivity, absolute temperature, and lattice and electron thermal conductivity, respectively.^2^ For convenience, *S*^2^σ is referred to as the *power factor*. Generally, the strategies for enhancing the TE performance of a material are based on improving charge transport behavior (increasing *S* and/or σ) and/or suppressing thermal transport (reducing *k*_l_ + *k*_e_). While established commercial TE materials are mainly based on Bi_2_Te_3_, the perovskite oxide strontium titanate has received increasing attention in recent years because of advantages offered in terms of chemical and thermal stability, lack of heavy elements, and nontoxic nature compared to traditional materials.^3^ Although undoped SrTiO_3_ is an insulator with a band gap of 3.2 eV,^4^ electron-doped SrTiO_3_ exhibits an impressive power factor of up to 3600 μW/mK^2^ at room temperature,^5^ comparable to that of state-of-the-art Bi_2_Te_3_ materials (near room temperature) and PbTe (500–800 K) materials.^5−7^ Unfortunately, the high thermal conductivity of SrTiO_3_ limits TE performance, with *zT* values well below unity.^6−8^ Consequently, there has been much effort to reduce thermal conductivity; techniques including reducing grain size and embedding nanoparticles have brought about a 20–50% reduction, yielding ∼3 W/mK at ∼1000 K.^8,9^ With the exception of a few studies that have rarely been replicated, the maximum *zT* values for strontium titanate are predominantly around 0.3–0.4 at 900–1000 K,^10−13^ while most state-of-the-art TE materials (such as those based on Bi_2_Te_3_ and PbTe) have *zT* > 1.0.^6,7^

Recently, studies on modulating the charge transport behavior of polycrystalline strontium titanate have focused on grain boundary (GB) engineering with synthesis under highly reducing conditions (e.g., carbon-encased samples during sintering in a reduced atmosphere) or graphene-based additions.^14−23^ A primary objective has been to reduce the GB resistance in polycrystalline SrTiO_3_, which suppresses charge transport, inhibiting electrical conductivity, especially at low temperatures.^24^ Double Schottky barriers are formed at the GBs^16^ by the depletion of positively charged oxygen vacancies near the GB, and these tend to filter low-energy electrons.^24^ Consequently, the carrier concentration and charge transport decrease at GBs. It was suggested that graphene-based materials located in GB regions can act as reducing agents, lowering the double Schottky barriers.^18,20^ Lin et al.^15^ exploited this concept and reported pseudo-single-crystal-like electron conductivity behavior at room temperature for strontium titanate-based samples containing 0.6 wt % graphene, achieving 2000 S/cm and a *zT* value of 0.4 at room temperature. Compared to graphene-free samples, electrical conductivity was enhanced by several orders of magnitude, especially at lower temperatures.^15^ Similar enhancement of the electrical conductivity in strontium titanate has been found for the incorporation of other graphene derivatives, including graphene oxide (GO) and reduced graphene oxide (rGO).^18−23^ However, the single-crystal-like charge transport behavior in strontium titanate has been most effective for additions of graphene-based materials.^15,17−19,23^

Here, we report an alternative approach to achieving single-crystal-like charge transport behavior and high carrier mobility at room temperature by the direct incorporation of molybdenum disulfide using a molecular precursor approach. The precursor (ammonium tetrathiomolybdate) decomposes to form molybdenum disulfide at a modest temperature of 360 °C.^25^ Molybdenum disulfide exhibits excellent carrier mobility of up to ∼410 cm/Vs at room temperature,^26−28^ which is comparable to or even higher than that of some graphene-based materials (e.g., rGO).^29^ Moreover, Chen et al.,^30^ when examining the thermal degradation of monolayer MoS_2_ on SrTiO_3_ supports, reported that molybdenum disulfide can absorb oxygen from strontium titanate and form MoO_*x*_ (*x* = 2 and 3) when heated in vacuum at 900 °C.^30^ During the processing of these SrTiO_3_-based materials, there will be a conversion from the precursor to MoS_2_, then to some form of Mo oxides, and ultimately to Mo particles. The initial conversion to form MoS_2_ is well established and may be described by
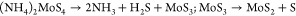


The precursor ammonium tetrathiomolybdate decomposes to MoS_3_ when heated to ≥120 °C in vacuum or inert gas; the continuous loss of sulfur leads to the formation of MoS_2_ flakes around 400 °C, which remain stable up to 900 °C.^25,31−33^ However, in the presence of even residual oxygen, MoS_2_ will be oxidized to MoO_*x*_ (*x* = 1.5, 2, and 3) at 360–900 °C.^30,33,34^ At the highest temperatures (>800 °C) and in a hydrogen atmosphere, the end product is metallic Mo^0^ particles.^35^ Thus, the precursor and its derivatives can promote GB engineering in strontium titanate and thereby enhance charge carriers and TE performance by mechanisms similar to those operating when graphene-based additives are employed.

We have chosen single-doped strontium titanate SrTi_0.85_Nb_0.15_O_3_ as a model system as it has been the subject of numerous studies,^19,23,36−39^ and while it does not exhibit the highest *zT* values (typically of up to 0.22–0.27 at 800 K^19,23,36,39^), it does allow us to isolate the effects of the additive more easily. In line with earlier studies, we employed 2 at % of TiO_2_, 1 at % of WO_3_, and 0.5 at % of V_2_O_5_ as sintering aids.^40−42^ We show that mixing SrTi_0.85_Nb_0.15_O_3_ powders with ammonium tetrathiomolybdate enables single-crystal-like charge transport behavior to be achieved directly after sintering without any additional heat treatment. This provides a much simpler and more efficient method to attain high charge mobility, high electrical conductivity, and high power factor in polycrystalline oxide titanates for TE applications.

## 2 Experimental Methods

### 2.1 Starting Materials

SrTi_0.85_Nb_0.15_O_3_ (denoted as N15) was prepared via solid-state reaction. Starting materials were powders of SrCO_3_, TiO_2_, and Nb_2_O_5_ (all 99.9% grade from Sigma-Aldrich). Sintering additives were TiO_2_ (99.9%, Sigma-Aldrich), WO_3_ (99.995%, Sigma-Aldrich), and V_2_O_5_ (99.6%, Sigma-Aldrich). Ammonium tetrathiomolybdate was of 99.97% (Merck). Graphene nanoplatelets (used only as part of the sintering environment, denoted as GnPs, grade M25) were obtained from XG sciences.

### 2.2 Solid-State Synthesis

Powders of SrCO_3_, TiO_2_, and Nb_2_O_5_ were weighed according to the required stoichiometric ratio, then mixed with propan-2-ol and zirconia milling balls with a mass ratio of 1:1:1, and vibromilled for 24 h. After drying, the milled powders were calcined at 1473 K for 12 h in air. The as-prepared N15 powders were mixed with 2 at % of TiO_2_, 1 at % of WO_3_, and 0.5 at % of V_2_O_5_ (sintering additives^40−42^) and further milled in a planetary ball mill (PM100, Retsch) at 350 rpm for 4 h. The resulting N15 powders were added to propan-2-ol to form a suspension and tip sonicated (Sonics VCX 750 with a standard 1/2 inch diameter probe) for 0.5 h. Similarly, sufficient ammonium tetrathiomolybdate powder required to provide doping levels of 0.5, 1, and 2 wt % for the N15 powders was added to propan-2-ol and tip sonicated for 0.5 h. Finally, the two suspensions were mixed and tip sonicated for another 0.5 h, filtered, and dried. The resulting powders were uniaxially pressed in steel dies of 10 and 20 mm diameters at a pressure of 50 MPa. The discs were buried in open alumina crucibles containing N15 powder with 5% GnPs and sintered at 1700 K for 24 h in a tube furnace with a 5% H_2_/Ar atmosphere. The N15 samples containing 0, 0.5, 1, and 2 wt % ammonium tetrathiomolybdate are denoted as 0M, 0.5M, 1M, and 2M, respectively.

### 2.3 Characterization

Sample densities were determined by Archimedes’ method. Powder X-ray diffraction (XRD) analysis was undertaken by use of PANalytical X’Pert Pro with a Cu Kα source and a 2θ scan from 10° to 100° (0.03°/step and 320 s/step). X’Pert Highscore was used to identify the phases. The lattice parameters were obtained via Rietveld refinement using TOPAS version 5 software (Bruker AXS).^43^ Samples were ground and polished down to 0.25 μm silicon carbide. Surface morphology and compositions were examined by scanning electron microscopy [SEM, Tescan Mira3 equipped with energy-dispersive X-ray spectroscopy (EDS)] and an electron probe microanalyzer [JOEL FEG-EMPA equipped with wavelength-dispersive X-ray spectroscopy (WDS)]. Image J software^44^ was used to calculate the average grain size and volume fractions of phases. The average grain sizes were calculated via the linear intercept method on several SEM images of polished surfaces, while the volume fractions of phases were calculated via the pixel intensity (gray-scale) threshold ratio on several SEM images of polished surfaces. Secondary phase (SP) analyses were carried out by Raman spectroscopy using a Renishaw Invia 633 nm Raman microscope. The Raman data were calibrated against a standard silicon peak (Si 520.6 cm^–1^ Raman shift) and analyzed by Renishaw Wire 4.4.

X-ray photoelectron spectroscopy (XPS) was conducted using a Kratos Axis Ultra Hybrid XPS (Al Kα source, *E* = 1486.69 eV). The XPS data were calibrated by a C 1s (binding energy (BE) = 284.8 eV) peak and fitted by a mix of Lorentzian and Gaussian characteristics using CASA XPS software. Transmission electron microscopy (TEM) data were collected by the FEI Talos F200A scanning transmission electron microscope (STEM, equipped with a super-X energy-dispersive X-ray detector), operated at 200 kV; TEM samples were prepared by focused ion beam (FIB) techniques (FEI Helios Plasma FIB equipped with EBSD and EDS). Atomic force microscopy (AFM) data were collected using a Bruker MultiMode 8 powered by a NanoScope controller. Kelvin probe force microscopy (KPFM) data were collected by a MESP-V2 probe; the mode was amplitude modulated by KPFM with sample bias. The KPFM data were analyzed by Gwyddion.

Electrical conductivity and Seebeck coefficients were determined using an ULVAC ZEM-3 under a low-pressure He atmosphere (293–873 K). Thermal conductivity was obtained from the relationship: *k* = ρα*C*_p_, where ρ is the bulk density, α is the thermal diffusivity, and *C*_p_ is the specific heat capacity. Thermal diffusivity (samples were polished discs 6 mm in diameter and 1 mm thick) and specific heat capacity were determined from room temperature to 873 K in Ar using a Netzsch LFA-427 and Netzsch STA 449C, respectively.

## 3 Results and Discussion

The densities of all samples were high, above 94% theoretical, varying from 4.97 to 5.06 g/cm^3^ (Table 1). There was no significant change or systematic variation with sample doping. These densities are typically 3–5% higher than the Nb-doped strontium titanates reported by Okhay et al.,^19^ although the sintering temperature used in the present study was 140 K lower.^19^

**Table 1:** Sample Density, Percentage Theoretical Density, Average Grain Size, Concentration of Ti-Enriched SP, and Lattice Parameters

sample	density (g/cm^3^, ± 0.02 g/cm^3^)	% theoretical density (%, ±0.4%)	average grain size (μm, ±0.3 μm)	Ti-enriched SP (%, ± 0.2%)	lattice parameter^a^ (A)
0M	5.03	95.3	5.5	1.6	3.916013(4)
0.5M	5.06	95.8	5.7	2.6	3.926343(6)
1M	4.97	94.1	3.8	3.9	3.919953(7)
2M	5.03	95.3	3.6	3.7	3.916842(7)

a Numbers in parentheses show the uncertainty in the final digit.

**Figure 1:**
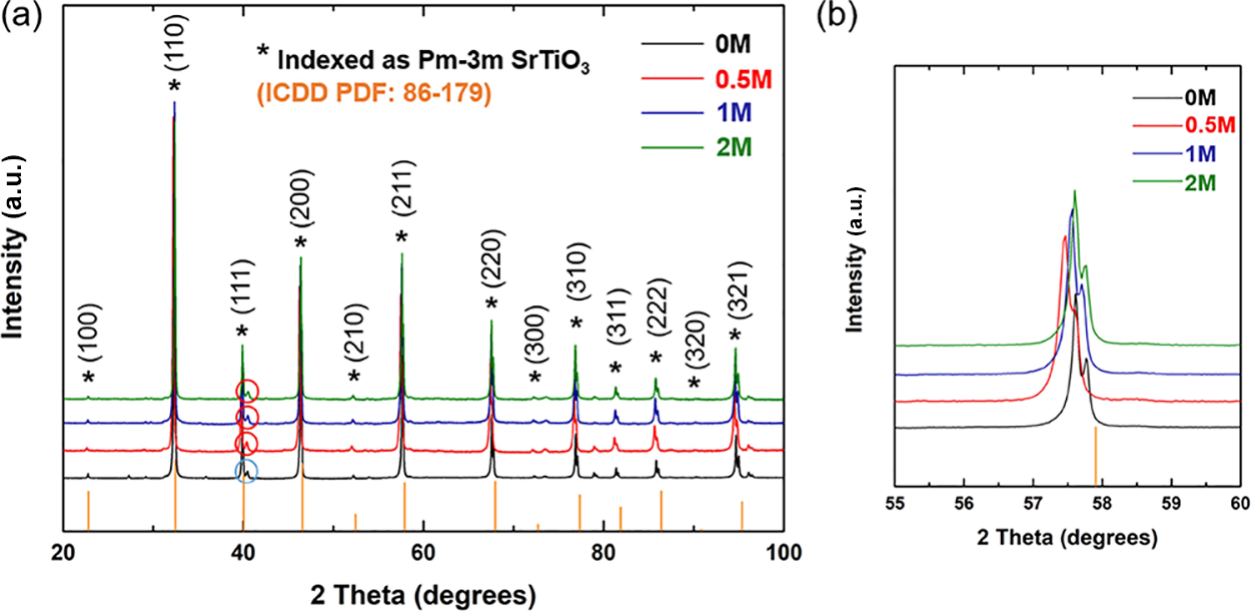
(a) XRD patterns at 2θ = 20°–100° for SrTi_0.85_Nb_0.15_O_3_-based samples (0, 0.5, 1, and 2M) after sintering. The blue circle shows the primary peak for metallic tungsten, and the red circles show the primary peaks for tungsten and molybdenum. (b) XRD patterns at 2θ = 55°–60° showing the peak positions for 0M, 0.5M, 1M, and 2M.

XRD patterns for the four samples (Figure 1) confirm that the primary phase can be indexed as a cubic perovskite (ICDD PDF Card: 86-179, space group: *Pm-3m*(45)) with minor peaks for SPs. Peaks associated with an SP were detected at 2θ = 40.5°. In samples of 0M, these peaks are believed to result from metallic tungsten (i.e., the sintering additive used), while in samples of 0.5, 1, and 2M, they come from a mixture of tungsten and molybdenum, the latter from the thermolysis of ammonium tetrathiomolybdate and subsequent reduction of the molybdenum(VI) and molybdenum(IV) sulfides.^46,47^ The presence of W and Mo in the samples was confirmed by WDS and EDS analyses (Figures S1 and S2). We note that although a 2% excess of TiO_2_ was added as a sintering aid, there is no evidence of any separate TiO_2_-based phase in the XRD patterns. However, upon the incorporation of ammonium tetrathiomolybdate (from 0M to *x*M, *x* = 0.5, 1, and 2), additional reflections associated with SrTiO_3_ (Figure 1b) appeared at low angles. This reflects the expansion of the lattice (Table 1) as a result of the generation of either additional oxygen vacancies or reduction of Ti^4+^ to Ti^3+^ (R_Ti_^4+^ = 60.5 pm and R_Ti_^3+^ = 67.0 pm).^48^ Furthermore, this indicates that ammonium tetrathiomolybdate, or more specifically the subsequent phases derived, can also act as a reducing agent in strontium titanate-based materials, in much the same way as graphene-based additives.^19^ The same synthesis conditions were used here for the 0M and *x*M (*x* = 0.5, 1, and 2) samples; the only difference is the amount of ammonium tetrathiomolybdate added. Moreover, in moving from 0.5M to 2M, the unit cell volume reduces; the reason remains unclear possibly due to a part of Mo occupying the Ti site in the perovskite structure (R_Mo_^6+^ = 59.0 pm and R_Ti_^4+^ = 60.5 pm).^49^

**Figure 2:**
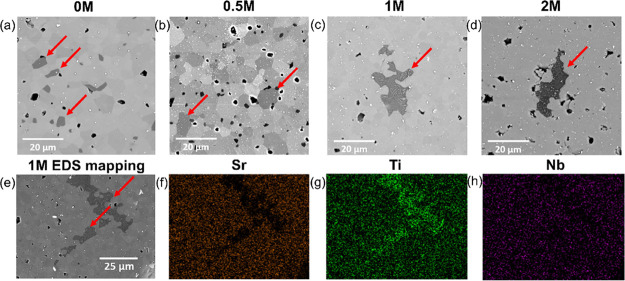
(a–d) Backscattered electron (BSE) images of four sintered samples, (a) 0M, (b) 0.5M, (c) 1M, and (d) 2M. (e-h) EDS maps for Sr, Ti, and Nb in the 1M sample: (e) BSE image of 1M, (f) Sr mapping, (g) Ti mapping, and (h) Nb mapping. The red arrows identify Ti-enriched SPs.

The microstructures and elemental distributions of the samples are shown in Figure 2. All microstructures contain polygonal grains with average sizes of typically 3.5–6 μm (Table 1); porosity (1–2 μm in size) is visible at some of the triple points. The darker grains in the microstructures (Figure 2a–e) are SPs, being rich in Ti, but there is no evidence of Sr or Nb (Ti-enriched SP); they vary in size and shape from polygonal and several microns across in 0M and 0.5M (with compositions close to TiO_2_ (Figure S3)) to irregular in shape and tens of microns in size in 1M and 2M (with compositions close to Ti_2_O_3_ (Figure S3)). The white-color SP particles in 0M (Figure 2a,e) are metallic tungsten, resulting from the sintering additives, and in *x*M (*x* = 0.5, 1, and 2), the particles are a mixture of tungsten and molybdenum from the precursor ammonium tetrathiomolybdate (see Figures S1 and S2). The size of the white-color SP particles increased with the amount of the precursor in the starting mixture, reflecting the introduction and agglomeration of Mo into the microstructure.

In samples of 0M and 0.5M, the grain sizes were ∼5.5 μm and are typically ∼50% larger than 1M and 2M samples (∼3.8 μm), suggesting that the precursor, when present at levels of ≥1 wt %, acts as a grain growth inhibitor, similar to the behavior of rGO in strontium titanate.^19^ On the other hand, the amount of Ti-enriched SP increased systematically with the amount of ammonium tetrathiomolybdate in the starting mixtures (Table 1), reaching a maximum of 3.7% in 2M samples. Thus, ammonium tetrathiomolybdate, or the subsequent phases derived, appears to have the same effect as graphene-based materials in stimulating the precipitation of titanium-rich oxides from the strontium titanate matrix.^50^ Furthermore, as shown in Figure S4, the Ti-enriched SP grains in 1M and 2M samples are large and distributed relatively uniformly, suggesting that the ammonium tetrathiomolybdate precursor is uniformly distributed within the strontium titanate during processing.^50^

EDS mapping (Figure S5) suggests that the solubility of Nb in the Ti-enriched SP reduced significantly when the precursor content in the starting mixes was ≥1 wt %. Most of the Ti-enriched SPs in the 0M and 0.5M samples are rich in Nb, while most SPs in 1M and 2M samples are deficient in Nb. These results contrast with the work of Li et al.^50^ for rGO-incorporated, Nb-doped strontium titanate; they reported that rGO promoted precipitation of Nb in the Ti-enriched SP.^50^ These differences can be explained by the high solubility of Nb in TiO_2_^51^ and the higher Sr content in the present work [Sr/(Ti + Nb) = 0.97] compared to that in Li et al.’s work [Sr/(Nb + Ti) = 0.91].^50^ The relatively high Sr content in the present work (but slightly deficient compared to stoichiometric SrTiO_3_) leads to the formation of a TiO_2_ SP in the standard N15 sample, while in Li et al.’s work, the low Sr content helped to form Ti_3_O_5_. The addition of ammonium tetrathiomolybdate helps in reduction of TiO_2_ to Ti_2_O_3_ (in contrast, Ti_3_O_5_ transformed into TiO_2_ with the addition of rGO in Li et al.’s work). It is inferred that the solubility of Nb is lower in Ti_2_O_3_ than in the matrix SrTiO_3_ and much lower than that in TiO_2_.

**Figure 3:**
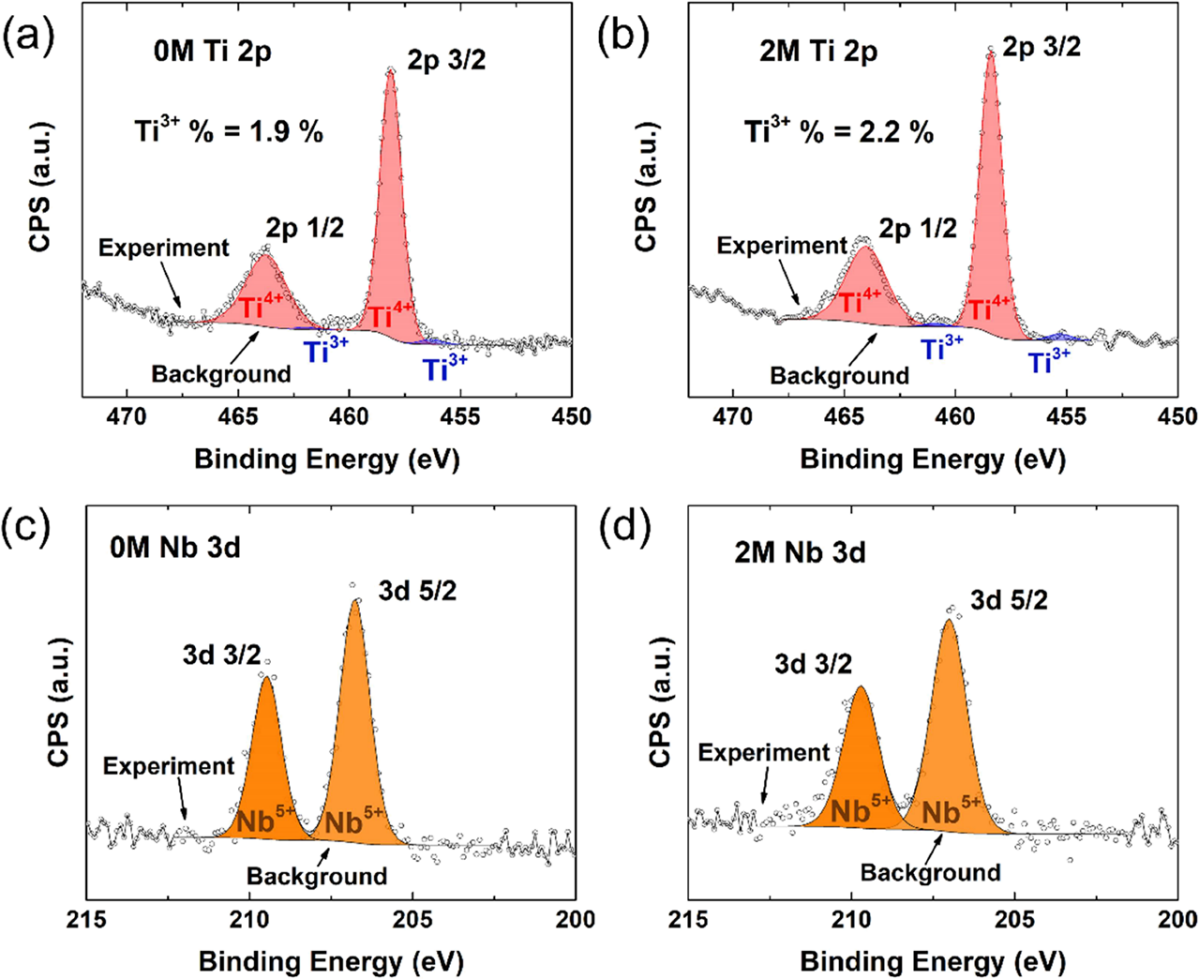
High-resolution XPS spectra (counts per seconds, CPS, vs binding energy) for 0M and 2M samples: Ti 2p collected from (a) 0M and (b) 2M samples; Nb 3d collected from (c) 0M and (d) 2M.

To identify the Ti and Nb valence states in the samples, high-resolution XPS spectra for Ti 2p and Nb 3d were collected from 0M and 2M (Figure 3). The Ti 2p orbital splits into Ti 2p_1/2_ and Ti 2p_3/2_ core levels, and both samples have a spin orbital splitting energy of approximately 5.7 eV, consistent with earlier investigations.^13^ Similarly, the Ti^4+^ binding energy peaks for the two samples [∼ 458 eV (2p_3/2_) and ∼ 464 eV (2p_1/2_)] match the previously reported Ti 2p energy data.^13^ The calculated concentrations of Ti^3+^ are 1.9% in 0M and 2.2% in 2M (hence, [Ti^3+^]_0M_/[Ti^3+^]_2M_ ≈ 0.864). As the carrier concentration (*n*) is related to [Ti^3+^],^52^ the [Ti^3+^] data suggest that the carrier concentrations in these two samples are comparable and relatively unaffected by the incorporation of ammonium tetrathiomolybdate. The Nb 3d XPS data for 0M and 2M show 3d_5/2_ and 3d_3/2_ peaks with a spin orbital splitting energy of ∼2.7 eV, similar to earlier Nb 3d data.^53^ Only Nb^5+^ peaks (∼207 eV for 3d_5/2_ and ∼209.7 eV for 3d_3/2_) were detected. Nb^5+^ is expected to form in Nb-doped SrTiO_3_ at Ti sites to provide donors.^54^ The lack of Nb^4+^ indicates that carriers are not localized by Nb in these samples.^55^

**Figure 4:**
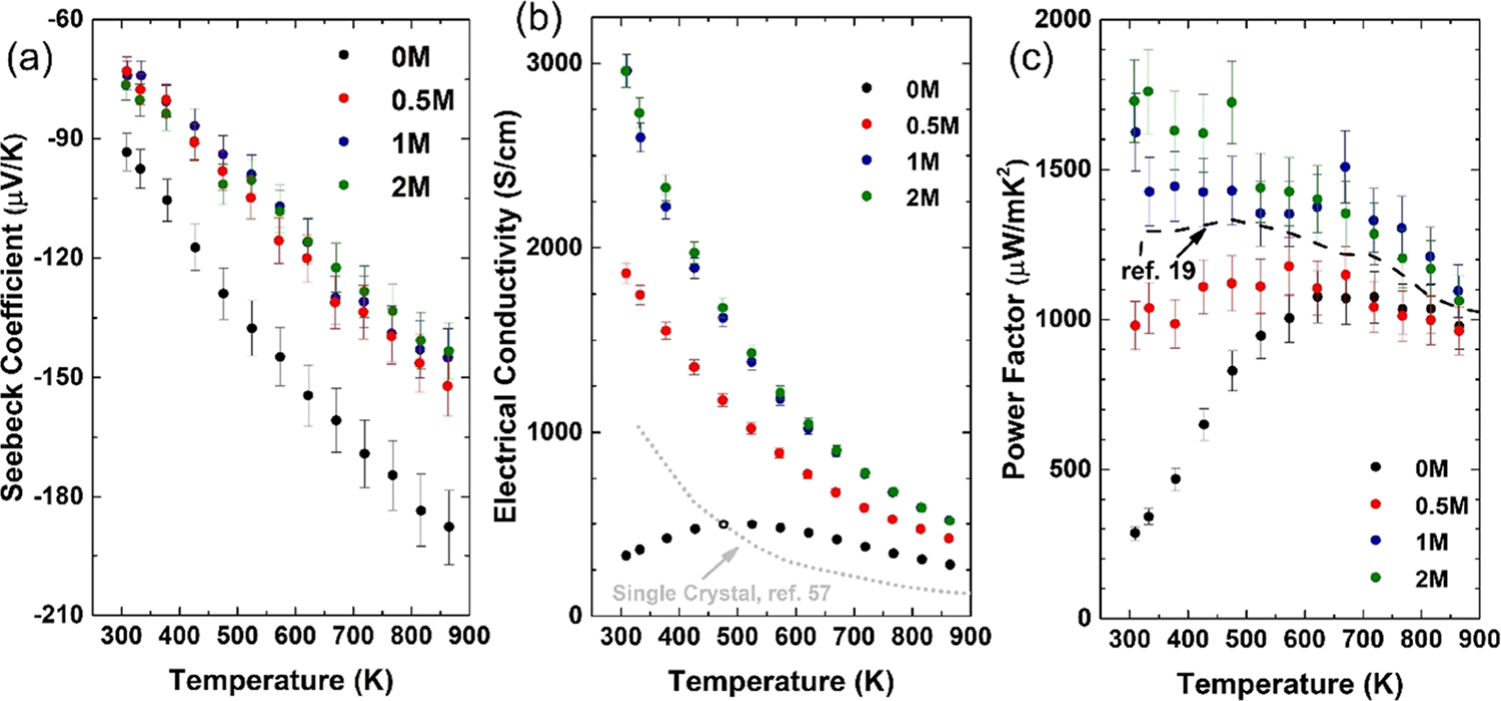
Charge transport properties of the 0M, 0.5M, 1M, and 2M samples. (a) Seebeck coefficients. (b) Electrical conductivity (the gray dotted line data are for single-crystal SrTiO_3_^57^). (c) Power factor values (note that the dashed line data are for a SrTiO_3_ composition (N15) containing 0.6 wt % rGO, from Okhay et al.^19^).

The charge transport properties of the samples are shown in Figure 4. The negative Seebeck coefficients (Figure 4a) indicate that all the samples are *n*-type. As anticipated, the 0M samples have higher |*S*| values (*S* = −93.3 to −187.8 μV/K), while the samples prepared with precursors 0.5M, 1M, and 2M have almost identical lower values of |*S*| (−73.0 to −152.1 μV/K). The electrical conductivity of 0M (Figure 4b) initially follows a similar trend to that for polycrystalline SrTiO_3,_^56^ increasing with temperature from 323 to 523 K (from 327 to 499 S/cm); the additional thermal energy enables more electrons to overcome the energy barriers. From 523 to 873 K, electrical conductivity decreases (from 499 to 277 S/cm) because scattering effects exceed the thermally enhanced transport of electrons at higher temperatures. While the three samples prepared with ammonium tetrathiomolybdate show pseudo-single-crystal trends (Figure 4b), similar to the single-crystal SrTiO_3_,^57^ the electrical conductivity decreases steadily with increasing temperature (room temperature to 873 K) from 2959 to 421 S/cm. The electrical conductivity of the 1M and 2M samples (∼ 2950 S/cm) is almost one order of magnitude higher than that of 0M at room temperature (327 S/cm) and ∼ 1.9 times higher (∼520 S/cm in 1M and 2M and 277 S/cm in 0M) at 873 K.

The power factor values for 0M are modest, increasing with temperature to a maximum of 1076 μW/mK^2^ at 623 K (Figure 4c). In contrast for samples prepared with ammonium tetrathiomolybdate, the considerably enhanced electrical conductivity combined with modest Seebeck coefficients leads to significant enhancement of the power factor in 1M and 2M, with a maximum of 1760 μW/mK^2^ at 323 K (Figure 4c). This is five times higher than that for 0M at 323 K and also 30% higher than that for polycrystalline SrTiO_3_ reported by Okhay et al.,^19^ which was prepared with the same level of Nb doping but containing 0.6 wt % rGO (N15–0.6rGO). Although the enhancement of the power factor reduces at high temperatures, 1M and 2M still exhibit power factors of ∼1200 μW/mK^2^ at 823 K, which is ∼20% higher than that for 0M and ∼ 10% higher than that for N15–0.6rGO.

To provide further information about the transport parameters, the carrier concentration and mobility were determined at ∼300 K from the Seebeck coefficients and electrical conductivity via the modified Heikes formula,^58^eqs 2 and 3. The effective mass of the electron m* was calculated from eq 4:^1^
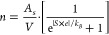
2
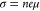
3
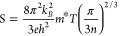
4where *n*, *V*, *S*, and σ refer to the carrier concentration, volume of the unit cell, Seebeck coefficient, and electrical conductivity, respectively; *e* is the electronic charge, *k_B_* is the Boltzmann constant, *A_s_* refers to the number of titanium sites in one unit cell,^59^ which is one in this case, and *h* is the Planck constant. It should be noted that spin and orbital degeneracy are negligible in this formula;^60^ therefore, the calculated results only provide estimates of the carrier concentration and mobility.^53^ The calculated values are presented in Table 2. The carrier concentrations are comparable in all four samples here (4.2–5.2 × 10^21^ cm^–3^_,_*n*_0M_/*n*_2M_ ≈ 0.857); the small variation in the carrier concentration is in accord with the fitting of XPS data for the Ti^3+^ concentration in 0M and 2M ([Ti^3+^]_0M_/[Ti^3+^]_2M_ ≈ 0.864). In contrast, carrier mobility increased significantly from 0.5 to 3.8 cm^2^/Vs with increasing amounts of the precursor in the starting mixtures. According to the Heikes formula (eqs 2 and 3), the higher carrier mobility in *x*M (*x* = 0.5, 1, and 2) will increase electrical conductivity without degrading the Seebeck coefficients, thus enhancing the power factor from 0M to 2M (Figure 4c). Moreover, the large effective mass values (m* = 10.2 ± 0.7 *m*_0_) for all four samples are comparable with previous values reported for Nb-doped strontium titanate.^57,61,62^ Since the effective mass can be directly derived from, and reflects, the electronic band structure of the materials,^62^ the close similarity in the effective mass values (Figure S6) for the present samples suggests that the physical properties of the matrix were not strongly affected by the use of the precursor. It is inferred (and will be justified later) that the use of the ammonium tetrathiomolybdate precursor modified the GB regions of strontium titanate.

**Table 2:** Transport Properties of Four Samples at Room Temperature^a^^,^^b^

sample	*S* (μV/K)	σ (S/cm)	*S*^2^σ (μW/mK^2^)	*n* (10^21^ cm^–3^)	μ (cm/Vs)	m*/*m*_o_
0M	–93.3	327	285	4.2	0.5	10.9
0.5M	–73.0	1861	992	5.0	2.3	9.6
1M	–74.1	2962	1628	5.2	3.6	10.0
2M	–76.4	2958	1727	4.9	3.8	10.0

a Seebeck coefficient (*S*), electrical conductivity (σ), power factor (*S*^2^σ), carrier concentration (*n*), carrier mobility (μ), and effective mass (m*/*m*_0_; *m*_0_, electron rest mass); the last three have been calculated via eqs 2 to 4.

b Uncertainties are 5, 3, 8, 10, 10, and 10% for S, σ, S^2^σ, *n*, μ, and m*/*m*_0_, respectively.

**Figure 5:**
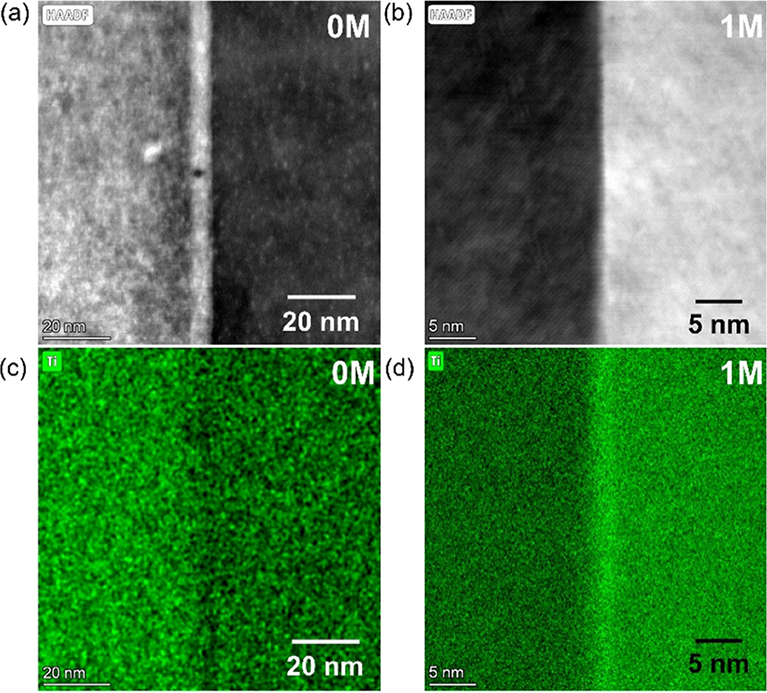
STEM high-angle annular dark field (HAADF) images of GB regions in (a) 0M sample and (b) 1M sample and the corresponding Ti EDS maps for (c) 0M sample and (d) 1M sample.

To understand the critical changes occurring in the GB regions, samples 0M and 1M were examined by STEM–EDS (Figure 5). It is clear that there are significant chemical differences between the two samples; EDS mapping shows that the GBs are depleted in Ti in 0M samples (Figure 5c) but enhanced in Ti in 1M samples (Figure 5d). More detailed EDS maps for the two samples (Figure S7) confirm that there was very limited segregation or modification of the “base” samples (0M): slight depletion of Ti and possibly Sr but slight enhancement of Nb. This suggests that the concentration of oxygen vacancies in the boundary regions would be modest because of the enrichment of Nb, giving rise to a significant GB barrier. In contrast, EDS maps for the GB region in sample 1M (Figure S7) reveal the depletion of Sr and Nb but enhancement of Ti. Previous studies^52,63^ indicate that both Ti^3+^ and Ti^4+^ species are expected; from the depletion of Nb, it is inferred that Nb has high solubility in Ti^4+^-rich phases but possibly insoluble in Ti^3+^-rich phases, i.e., the GB being rich in Ti^3+^. Thus, the heavy depletion of Nb^5+^ would lead to enhancement of oxygen vacancies in the GB region, and the increase of Ti^3+^ would introduce more carriers^52^ in 1M samples. Together, this would cause a reduction in the GB barrier and enhanced electrical conductivity.

**Figure 6:**
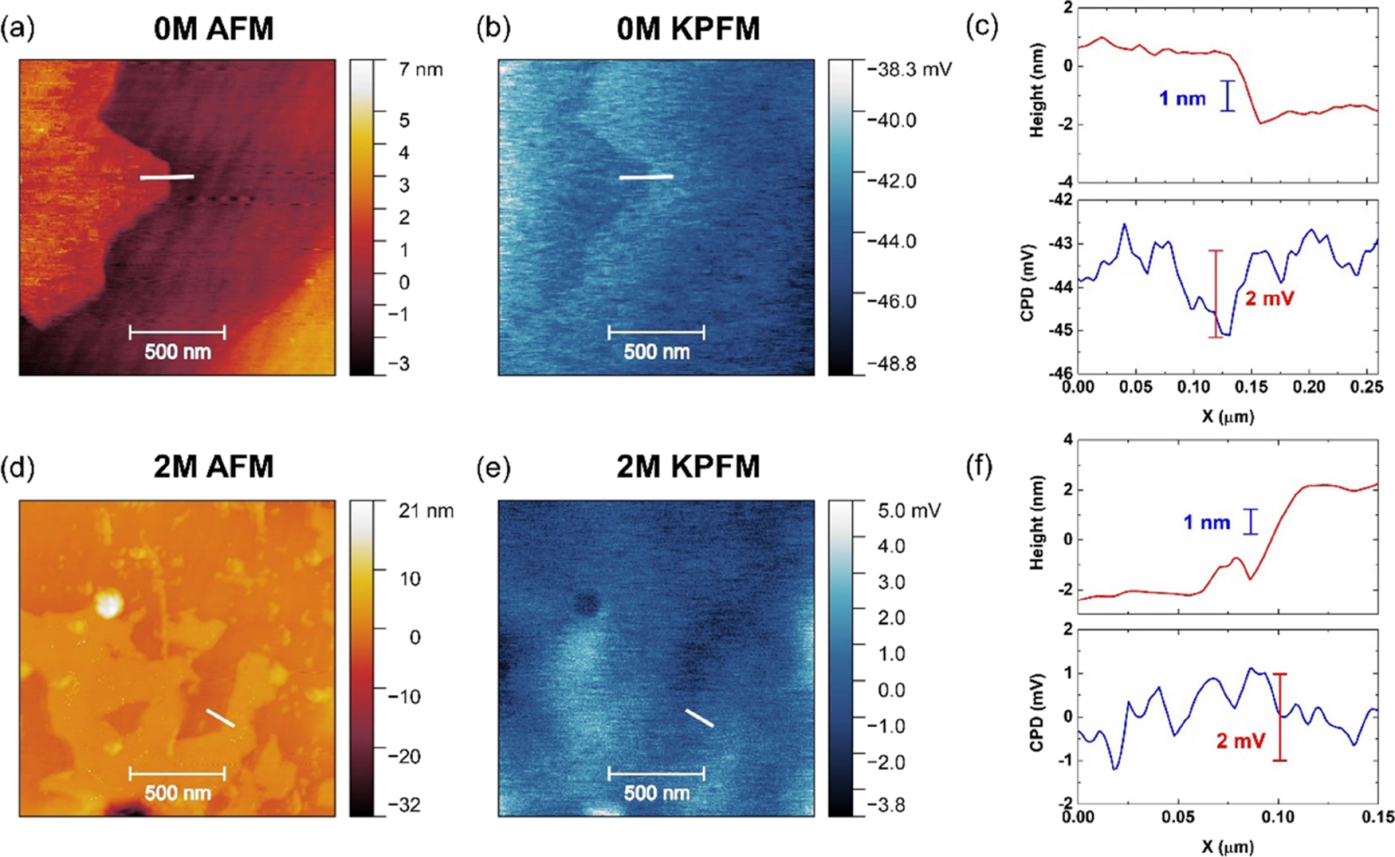
AFM topography and corresponding Kelvin probe (KPFM) scans and the line profiles for the 0M sample (a–c) and the 2M sample (d–f).

In view of the similarity of the transport behavior of samples 1M and 2M (Figure 4 and Table 2), we selected 2M (representative of high-conductivity samples) and 0M (highly resistive) to investigate the nature of the GB barriers in the two types of samples. AFM topography and Kelvin probe (scanning surface potential microscopy) data are presented in Figure 6. Each sample contains at least one clear GB in the region scanned. The AFM data (Figure 6a,d) were collected at the same time as contact potential difference (CPD) data from the Kelvin probe (Figure 6b,e); these enabled CPD line profiles and heights across the GB regions to be extracted (Figure 6c,f). The AFM profiles for both samples indicate changes in height ∼2 nm across the GB; this is comparable with reported AFM data for GB regions in SrTiO_3_-based materials.^14^ In contrast, the CPD line profiles are markedly different. The 0M sample shows a clear “valley” structure, with a change in potential in excess of 2 mV, suggesting the existence of negatively charged GB potentials,^14,24^ while the CPD profiles for 2M samples are much reduced in size (well under 2 mV), indicating much lower potential barriers in samples that have been prepared with the precursor.

**Figure 7:**
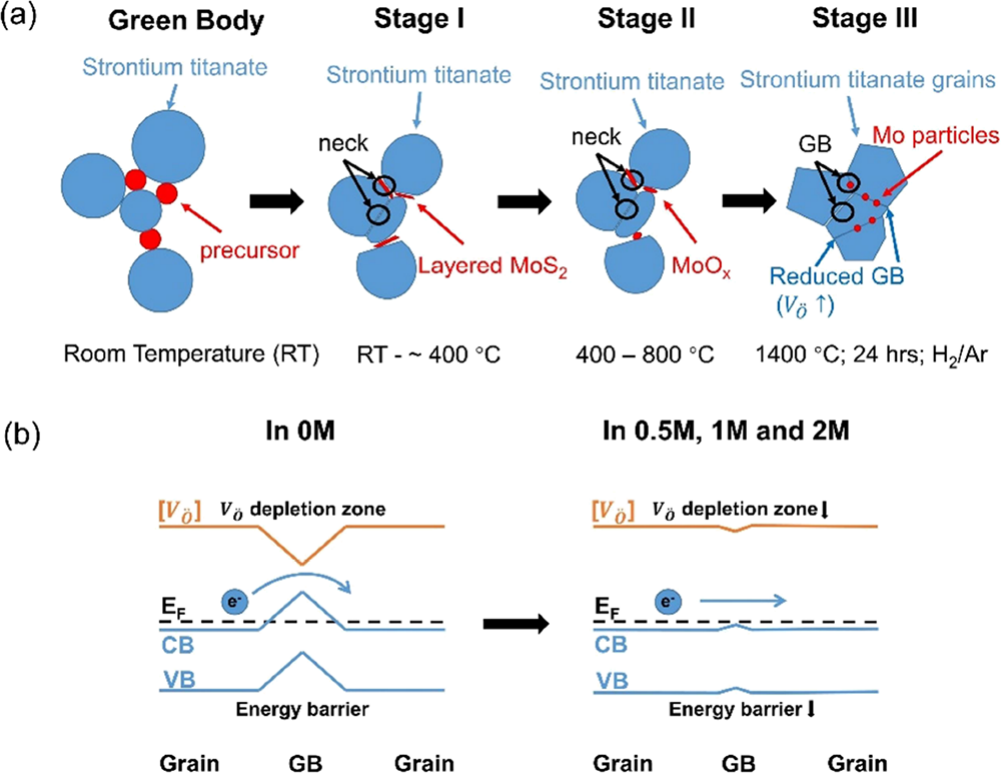
Schematic diagrams showing (a) process for forming MoS_2_ and its conversion to Mo at the GBs during sintering and (b) principle of lowering the GB barrier to achieve high and single-crystal-like electrical conductivity in going from 0M to 0.5M/1M/2M samples.

The processes for forming MoS_2_ and its conversion to Mo at the GBs during sintering are shown schematically in Figure 7. The initial process step results in the deposition of the precursor between the strontium titanate grains (Figure 7a). During Stage I of sintering (temperatures up to 400 °C), necks begin to form between the grains, and the precursor is converted to MoS_2_ (Figure 7b).^25,31−33^ With increasing temperature, Stage II of sintering (400–800 °C), oxygen is absorbed from the GB regions, and MoS_2_ is converted to MoO_*x*_ (*x* = 1.5, 2, and 3);^30,33,34^ the generation of oxygen vacancies in the strontium titanate GBs leaves heavily reduced SrTiO_3−δ_. At the highest temperatures, Stage III of sintering (∼ 800–1400 °C), MoO_*x*_ (*x* = 1.5, 2, and 3) are converted into metallic Mo particles.^35^ These steps can be summarized as

Stage I: (NH_4_)_2_MoS_4_ → 2NH_3_ ↑ + H_2_S ↑ + MoS_2_ + S ↑ (∼400 °C);
Stage II: MoS_2_ + SrTiO_3_ (GB) → MoO_*x*_ (*x* = 1.5, 2, 3) + SrTiO_3−δ_ (GB) + S ↑ (∼ 400–800 °C);
Stage III: MoO_*x*_ (in H_2_/Ar) → Mo + H_2_O ↑ (∼800 to 1400 °C).

The exsolution of metallic Mo particles in Stage III is believed to have limited impact on electrical performances of strontium titanate, as reported by Kovalevsky et al.^64^ Increased concentrations of oxygen vacancies in Stage II result in enhanced carrier concentrations and weakening of the depletion zones in GB regions. Thus, the detrimental characteristics of highly resistive GBs with large potential barriers and low electrical conductivity in 0M samples are overcome and almost eliminated by the use of the MoS_2_ precursor, leading to increased carrier mobility and single-crystal-like carrier transport behavior (Figure 7b). Therefore, additions of ammonium tetrathiomolybdate (and its conversion to MoS_2_, MoO_*x*_, and finally Mo) appear to play a similar role to graphene in strontium titanate to modify the GB regions.^15,18−20^ This precursor approach also offers many advantages in terms of a much simpler processing route, avoiding complicated synthesis steps required for high-quality graphene and eliminating the need for the inclusion of an expensive nanomaterial.

**Figure 8:**
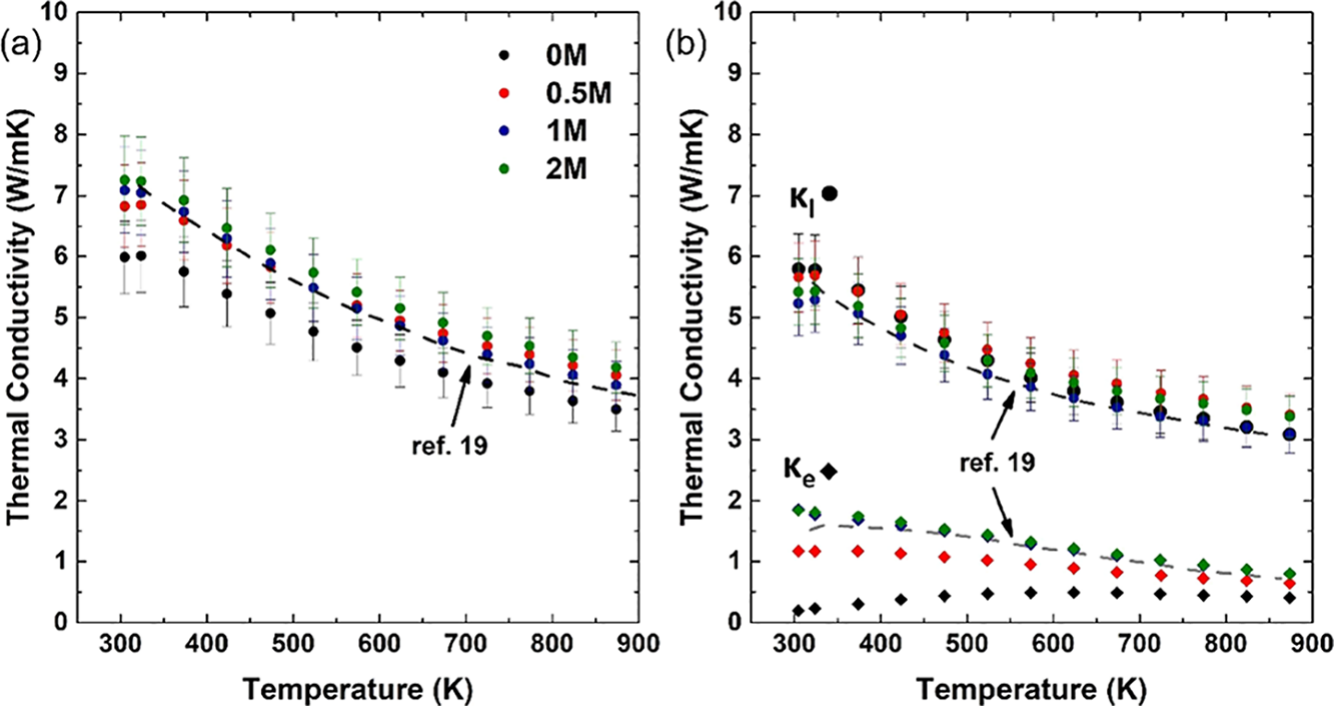
(a) Thermal conductivity and (b) lattice and electronic thermal conductivity for the four samples and the N15–0.6rGO sample from Okhay et al.^19^

Total thermal conductivity data (*k*) for 0 to 2M (Figure 8a) show very similar trends, with *k* decreasing with increasing temperature but increasing with the amount of the precursor added to the samples. Indeed, values for 0.5M, 1M, and 2M are very close to data reported for N15–0.6rGO^19^ but about 10% higher than that for 0M (Figure 8a). Globally, total thermal conductivity values range from 7.25 W/mK at room temperature to 3.5 W/mK at 873 K, comparable to that in studies of similar strontium titanate ceramics.^36^

The electronic thermal conductivity *k*_e_ can be calculated via the Wiedemann–Franz law *k*_e_ = *L*σ*T*, where *L* is the Lorenz number that can be obtained from Seebeck coefficients *S* (Figure S8). Values of *k*_e_ and *k*_l_ for the different samples are presented in Figure 8b. At room temperature, *k*_e_ constitutes almost 30% of the total thermal conductivity in 1M and 2M, comparable to the graphene-containing N15–0.6rGO SrTiO_3_,^19^ while in the reference sample 0M, *k*_e_ is only ∼3.7% of the total thermal conductivity. At high temperatures (∼850 K), the difference between the contributions is reduced, and *k*_e_ is typically 23% of the total thermal conductivity. The phonon mean free path can be estimated by the Debye–Callaway model:
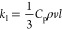
5
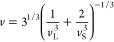
6where *C*_p_ is the specific heat capacity, ρ is the density, *v* is the average sound velocity, l is the phonon mean free path, and *v*_L_ and *v*_S_ are the longitudinal and transverse sound velocities.^8,55^ It is found that *v*_L_ and *v*_S_ for strontium titanate are relatively unaffected by the type of doping; the average sound velocity is approximately 5300 m/s.^65^ The calculated phonon mean free path values are around 1.1–1.2 nm (Table S1). Consequently, microscale features, such as changes of grain sizes, will have limited influence on the nanoscale phonon mean free path and scattering processes. Furthermore, the XPS data indicated only small changes in Ti^3+^ concentrations across the range of samples, leading to a comparable point defect concentration. Moreover, the observed Mo particles are mostly submicron in size with some less than 100 nm. Hence, it is possible that Mo particles act as nanoinclusions and scatter phonons.^64^ Such an enhanced scattering effect may offset or overcome the detrimental high thermal conductivity imparted to the system by metallic Mo. Nevertheless, there is a small reduction (∼ 8%) in the calculated phonon mean free paths in going from 0M to 1M and 2M (Table S1).

**Figure 9:**
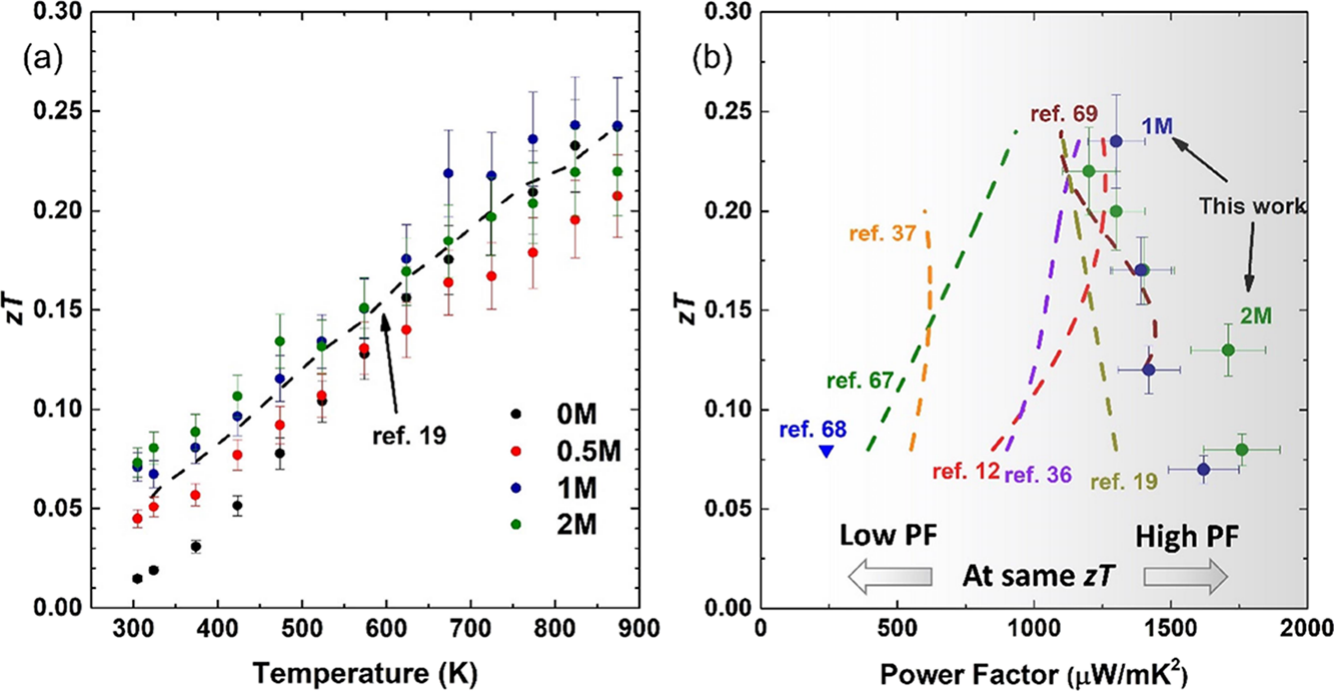
(a) *zT* values for the 0, 0.5, 1, and 2M samples as a function of temperature, compared with data for the N15–0.6rGO sample (dashed line, from Okhay et al.^19^). (b) Comparison of power factor values for different Nb-doped strontium titanate materials^12,19,36,37,67−69^ and their dependence on the *zT* value. Note that the data points located at the far right side of the figure (gray part) exhibit the highest power factors when comparing samples having the same *zT* values.

Based on the combined transport properties (Figures 4 and 8), the dimensionless TE figure-of-merit *zT* values were calculated (Figure 9a). The greatly enhanced electrical conductivity in 1M and 2M samples results in a fourfold increase in *zT* values at room temperature (∼0.07) compared to the reference 0M samples. Above 625 K, the differences between figure-of-merit values are much reduced as electrical conductivity values become much closer for all types of samples. The maximum *zT* value of ∼0.24 at 823 K was achieved for 1M samples; this is higher than the value reported for equivalent graphene-containing samples [N15–0.6rGO^19^ at the same temperature (∼0.22)]. Although higher *zT* values have been reported in a number of Nb-doped SrTiO_3_ (∼ 0.3 at 823 K),^12,36,39^ it is worth noting that all the present samples (0.5M, 1M, and 2M) have excellent power factor values (up to 1760 μW/mK^2^) at temperatures of up to 873 K (Figure 4c). For device applications, a high power factor is generally more important than maximum *zT*; it is preferable to have pairs of high power factor materials with comparable *zT* values as the output power is directly related to the power factor.^13,66^ In order to compare the maximum power factor and *zT* values simultaneously, we have combined the data in Figure 9b; the *x*M (*x* = 1 and 2) samples have higher power factors at *zT* = 0.08–0.24 when compared to other high-*zT* Nb-doped strontium titanates.^12,19,36,37,67−69^ The data points for *x*M (*x* = 1 and 2) samples are located at the far right side of Figure 9b, suggesting that a TE device based on *x*M (*x* = 1 and 2) materials should be able to generate more power than other samples with the same *zT* values. In general, our processing approach provides a much more direct and simpler way to deposit a two-dimensional material (here, MoS_2_) in the GB regions of strontium titanate compared to investigations employing graphene. Furthermore, the precursor/MoS_2_ approach yields materials with high power factors over a much wider temperature range, further increasing the operational window for SrTiO_3_ TEs.

## 4 Conclusions

High-quality SrTi_0.85_Nb_0.15_O_3_ TE ceramics, prepared with additions of 0–2 wt % ammonium tetrathiomolybdate (a precursor for MoS_2_), have been successfully synthesized via solid-state reaction in a reducing atmosphere. During processing, the precursor was converted to MoS_2_, then to MoO_*x*_, and finally to Mo particles, which were located in the GBs of SrTi_0.85_Nb_0.15_O_3_. Reactions occurring during processing led to the reduction of the matrix, and specifically GBs to SrTi_0.85_Nb_0.15_O_3-δ_, which enabled near-elimination of the resistive GB barriers.

The formation of MoS_2_, giving rise to Mo particles in the final microstructure, significantly increased carrier mobility but had limited impact on the carrier concentration. As a result, charge transport was enhanced and pseudo-single-crystal-like electrical conductivity occurred in the samples prepared with ammonium tetrathiomolybdate without degrading Seebeck coefficients. Compared to the reference samples (0M), the room-temperature electrical conductivity of the 1 and 2M samples increased by a factor of at least 8 (∼3000 S/cm); an exceptionally high power factor of 1760 μW/mK^2^ was obtained at room temperature. The maximum *zT* was 0.24 at 823 K.

The significantly improved electrical conductivity and power factor are particularly relevant for TE device applications. The addition of ammonium tetrathiomolybdate and its conversion to MoS_2_ and then to Mo was found to play a similar role to graphene in strontium titanate-based materials, helping the reduction of GB barriers. Importantly, our processing strategy provides a simpler and more direct way to achieve significantly enhanced charge transport behavior in strontium titanate and offers a possible route to “phonon-glass-electron-crystal” TE materials while avoiding the complex processing required in the synthesis of graphene-based materials.

## Supporting Information Available

Additional SEM images, EDS and WDS maps, and Raman spectra data of 0M, 0.5M, 1M, and 2M ceramics, STEM images and EDS maps of GB regions within the ceramics, calculated effective mass, and related thermal transport data (PDF)
