# Correction to “Precursor-Led Grain Boundary Engineering for Superior Thermoelectric Performance in Niobium Strontium Titanate”

**DOI:** 10.1021/acsami.3c03575

**Published:** 2023-03-20

**Authors:** Yibing Zhu, Feridoon Azough, Xiaodong Liu, Xiangli Zhong, Minghao Zhao, Kalliope Margaronis, Sohini Kar-Narayan, Ian Kinloch, David J. Lewis, Robert Freer

**Affiliations:** † Department of Materials, School of Natural Sciences, University of Manchester, Manchester M13 9PL, U.K.; ‡ Department of Chemistry, School of Natural Sciences, University of Manchester, Manchester M13 9PL, U.K.; § Department of Materials Science & Metallurgy, University of Cambridge, 27 Charles Babbage Road, Cambridge CB3 0FS, U.K.; ∥ Henry Royce Institute and National Graphene Institute, University of Manchester, Oxford Road, Manchester M13 9PL, U.K.

The published version of this paper listed only Robert Freer as the corresponding author. The purpose of this correction is to identify both David J. Lewis and Robert Freer as corresponding authors.

The conclusions of the work have not been affected by this correction.

