# Supplementary material for: Precursor-Led Grain Boundary Engineering for Superior Thermoelectric Performance in Niobium Strontium Titanate
Source: ACS Appl Mater Interfaces. 2023 Feb 28;15(10):13097–107. doi: 10.1021/acsami.2c22712 (PMC10020962; doi:10.1021/acsami.2c22712)
Supplement: Supplementary file 1 — am2c22712_si_001.pdf [file am2c22712_si_001.pdf]

Supporting Information

Precursor-led Grain Boundary Engineering for Superior Thermoelectric Performance in Niobium Strontium Titanate

Yibing Zhu[1]; Feridoon Azough[1]; Xiaodong Liu[1]; Xiangli Zhong[1]; Minghao Zhao[2]; Kalliope Margaronis[3]; Sohini Kar-Narayan[3]; Ian Kinloch[1,4]; David J Lewis[1]; Robert Freer[1]*

1. Department of Materials, School of Natural Sciences, University of Manchester, Manchester M13 9PL, United Kingdom
2. Department of Chemistry, School of Natural Sciences, University of Manchester, Manchester M13 9PL, United Kingdom
3. Department of Materials Science & Metallurgy, University of Cambridge, 27 Charles Babbage Road, Cambridge, CB3 0FS, United Kingdom
4. Henry Royce Institute and National Graphene Institute, University of Manchester, Oxford Road, M13 9PL, United Kingdom

*Corresponding Author:

robert.freer@manchester.ac.uk.

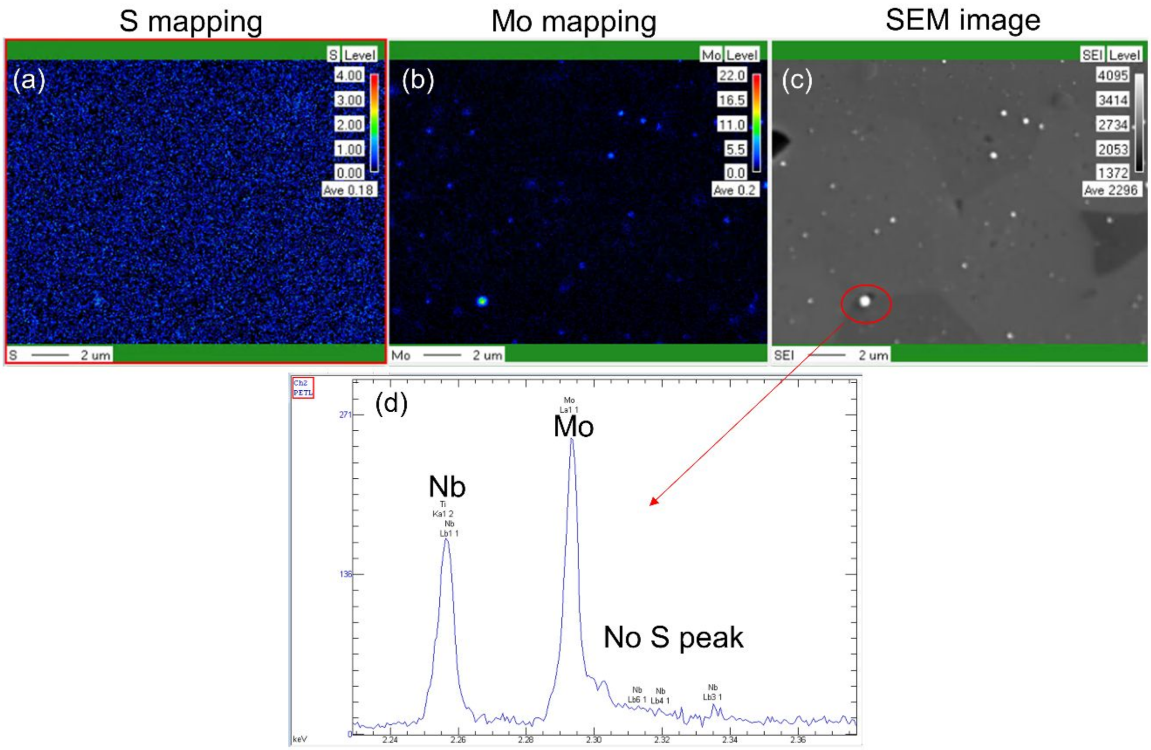

Figure S1. WDS maps and point analysis spectrum for 0.5M sample. (a): S map; (b): Mo map; (c): the corresponded SEM image; (d): spectrum collected from the circled particle identified in (c). The results indicate that the small white particles contain molybdenum but with no evidence of sulphur. By combining these data with the EDS results in Fig S2, the white particles in 0.5M, 1M and 2M samples can be confirmed to be metallic molybdenum or a mixture of metallic tungsten and molybdenum.

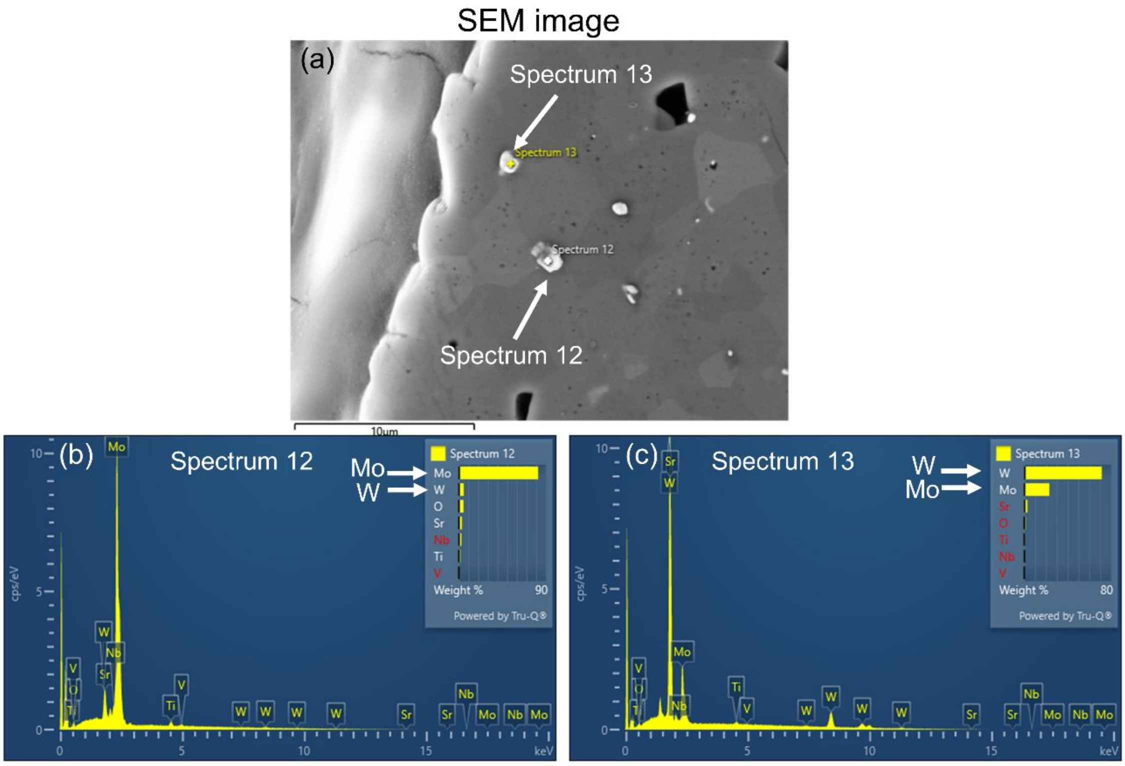

Figure S2. EDS analyses of points identified as 12 and 13 in 1M sample (a): SEM image; (b): EDS spectrum 12; (c): EDS spectrum 13. Combined with the WDS data (Fig S1), these results indicate that the white particles observed in the SEM image are either metallic tungsten, or molybdenum or a mixture of the two.

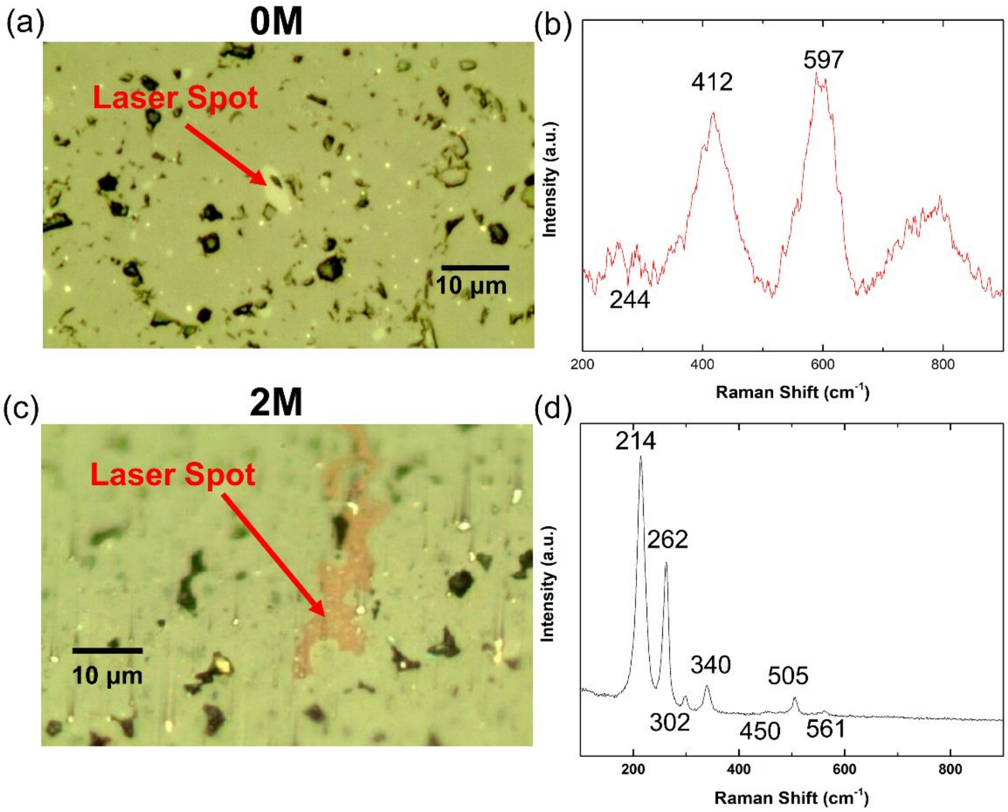

Figure S3. Optical microscope images and Raman spectra for the secondary phase in 0M sample (a) and (b), and 2M sample (c) and (d). These confirm that the secondary phase in the optical microscope images is TiO_2 [1] and in 2M is Ti_2O_3 [2].

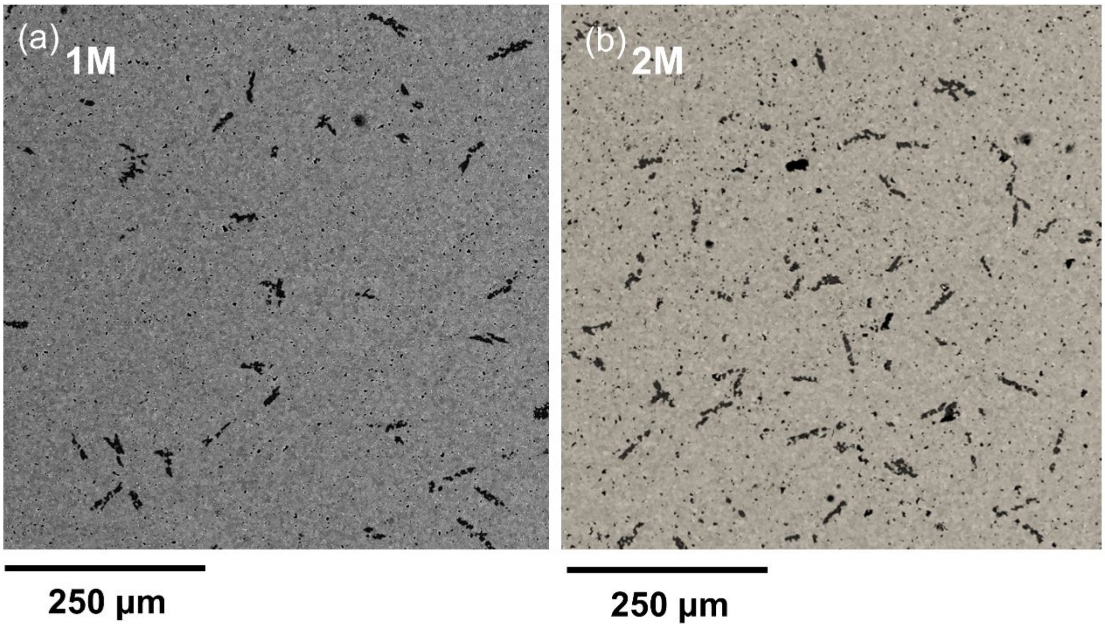

Figure S4. Low magnification BSE SEM images of (a) 1M and (b) 2M samples, showing the secondary phase precipitates (darker grains) are large and uniformly distributed within the matrix.

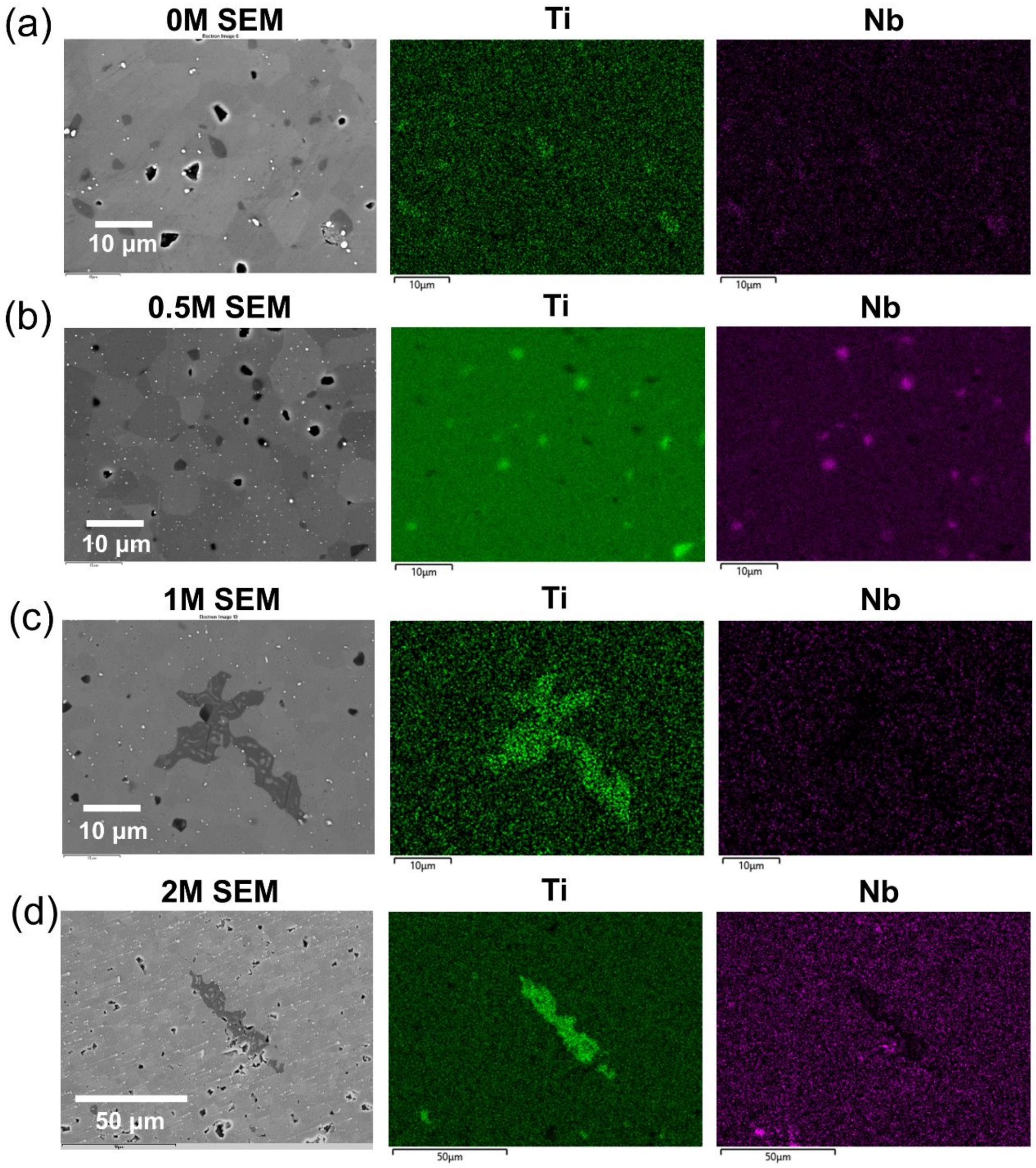

Figure S5. BSE SEM micrographs and SEM-EDS Nb and Ti elemental maps for: (a) 0M; (b) 0.5M; (c) 1M; (d) 2M samples.

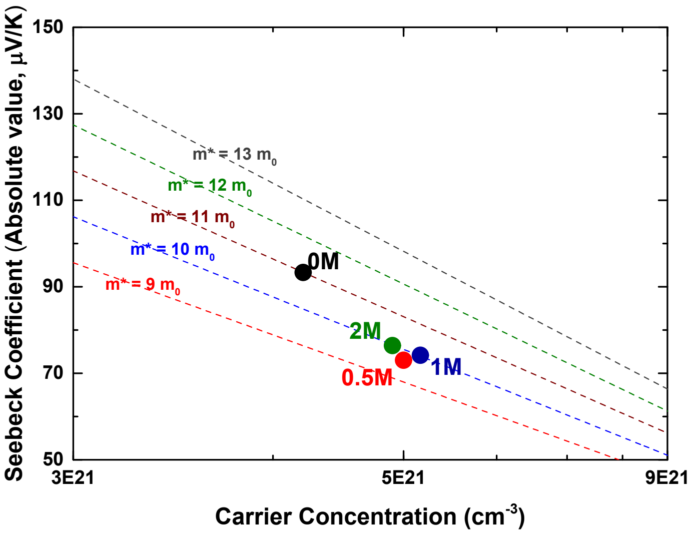

Figure S6. Seebeck coefficients as a function of carrier concentration, showing calculated effective mass values (dotted lines with m* values).

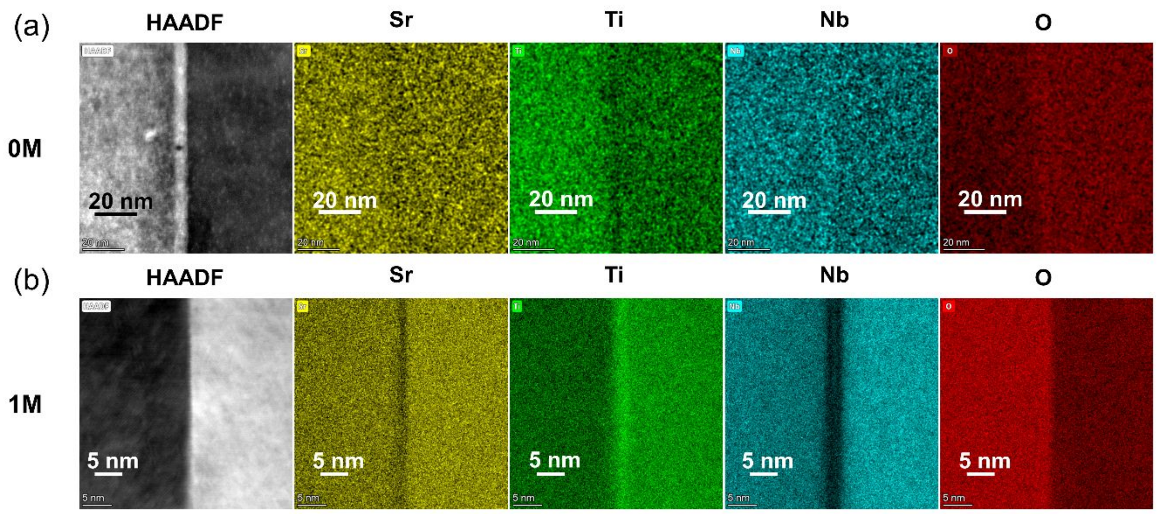

Figure S7. STEM-EDS data from the grain boundary regions of: (a) 0M and (b) 1M samples.

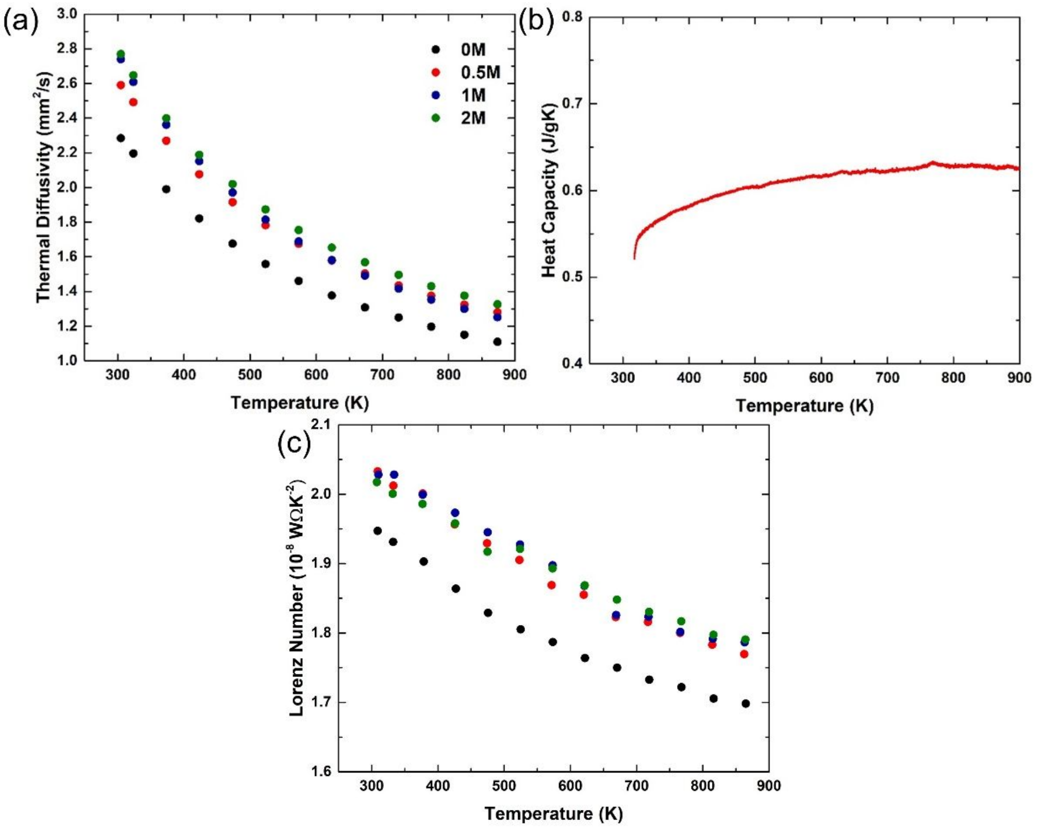

Figure S8. Temperature dependence of: (a) thermal diffusivity values for xM samples; (b) measured specific heat capacity for 0M sample; (c) Lorenz number calculated via $L = 1.5 + \exp[-|S|/116]$ (where S is Seebeck coefficient [3].

Table S1. Calculated phonon mean free paths (MPF) obtained via the Debye-Callaway model.

Sample	Phonon MPF (nm)
0M	1.22
0.5M	1.20
1M	1.13
2M	1.14

References

1. Li, J.; Li, F.; Zhuang, Y.; Jin, L.; Wang, L.; Wei, X.; Xu, Z.; Zhang, S., Microstructure and dielectric properties of (Nb+In) co-doped rutile TiO_2 ceramics. *J. Appl. Phys.* **2014,** *116* (7), 074105-074113.

2. Shvets, P. V.; Caffrey, D.; Fleischer, K.; Shvets, I.; O'Neill, K.; Duesberg, G. S.; Vinichenko, A. N.; Maksimova, K. Y.; Goikhman, A. Y., Suppression of the metal-insulator transition in magnetron sputtered Ti_2O_3 films. *Thin Solid Films* **2020,** *694*, 137642-137647.
3. Kim, H.-S.; Gibbs, Z. M.; Tang, Y.; Wang, H.; Snyder, G. J., Characterization of Lorenz number with Seebeck coefficient measurement. *APL Mater.* **2015,** *3* (4), 041506-041510.
